# Pareto Front Transformation in the Decision-Making Process for Spectral and Energy Efficiency Trade-Off in Massive MIMO Systems †

**DOI:** 10.3390/s25051451

**Published:** 2025-02-27

**Authors:** Eni Haxhiraj, Desar Shahu, Elson Agastra

**Affiliations:** Faculty of Information Technology, Polytechnic University of Tirana, 1001 Tirana, Albania; dshahu@fti.edu.al (D.S.); eagastra@fti.edu.al (E.A.)

**Keywords:** spectral efficiency, energy efficiency, optimization, decision-making, pareto front, Box–Cox transformation

## Abstract

This paper presents a method of choosing a single solution in the Pareto Optimal Front of the multi-objective problem of the spectral and energy efficiency trade-off in Massive MIMO (Multiple Input, Multiple Output) systems. It proposes the transformation of the group of non-dominated alternatives using the Box–Cox transformation with values of λ < 1 so that the graph with a complex shape is transformed into a concave graph. The Box–Cox transformation solves the selection bias shown by the decision-making algorithms in the non-concave part of the Pareto Front. After the transformation, four different MCDM (Multi-Criteria Decision-Making) algorithms were implemented and compared: SAW (Simple Additive Weighting), TOPSIS (Technique for Order of Preference by Similarity to Ideal Solution), PROMITHEE (Preference Ranking Organization Method for Enrichment Evaluations) and VIKOR (Vlse Kriterijumska Optimizacija Kompromisno Resenje). The simulations showed that the best value of the λ parameter is 0, and the MCDM algorithms which explore the Pareto Front completely for different values of weights of the objectives are VIKOR as well as SAW and TOPSIS when they include the Max–Min normalization technique.

## 1. Introduction

Mobile communication is one of the fastest-evolving technologies, with its fifth (5G) generation currently being implemented and its sixth (6G) generation already being studied and conceptualized. This evolution comes as a result of the continuously growing number of devices connected to mobile networks. Not only is the number of mobile users increasing rapidly, but mobile communication is also being introduced in new areas of application. Therefore, data traffic has been increasing rapidly and there is a strict demand for high transmission rates and low delays in communication. Massive Multiple Input, Multiple Output (MIMO) antenna systems are a critical technology that helps achieve the transmission requirements of today’s mobile communication networks. A Massive MIMO system comprises a large number of antennas, which can serve multiple users simultaneously on the same frequency band.

Considering that the frequency spectrum is limited, Massive MIMO should be able to serve more users on a limited frequency bandwidth, which means it should achieve high values of spectral efficiency. Spectral efficiency (SE) [bps/Hz] is the number of bits transmitted successfully per unit of time and bandwidth, and it can be increased by using a larger number of antennas and higher transmission power [1]. In the earlier generations of mobile communication, this was the primary focus of mobile operators. However, at present, the amount of energy consumed by a mobile network is also one of its most important aspects. The devices used for 5G base stations are more energy efficient, but the number of them that should be used to comply with the traffic being transmitted in the system is very high, and therefore the energy cost is also very high. A variety of different studies and estimations have predicted an excessive consumption of energy in 5G networks, which is damaging for the network users, for mobile operators who have the responsibility to cover the financial cost, and also for the environment, whose protection has become an emergency in our society [2,3]. Thus, the mobile communication systems of the future are required to guarantee not only a high spectral efficiency, but also a high energy efficiency (EE) [bps/J], which can be defined as the number of bits successfully transmitted per unit of time and energy. Evidently, these two objectives contradict each other, so their simultaneous maximization is a multi-objective optimization problem [4].

Much previous research has been conducted on this problem, mainly focusing on using different evolutionary multi-objective optimization algorithms to solve it [5]. The optimization algorithms generate a Pareto Optimal Front, comprised of non-dominated solutions, which means that none of the points in the Pareto Front (PF) is better than another regarding both optimization objectives [6]. Also, it has been proven that different optimization algorithms have similar results, so they generate Pareto Fronts close to the ideal one when they are used with a high number of generations and a high number of individuals per generation [6,7]. Previous work focused on each efficiency separately has shown that the maximization of energy efficiency is a non-convex issue, which means that it achieves a maximum value and then begins to decrease with further increase in the values of the variables [8]. This non-convex optimization issue is more complicated, and it requires special methods to be solved, such as the ones proposed in refs. [9,10]. Taking into consideration this fact, the multi-objective optimization problem regarding the trade-off between two efficiencies is also a non-convex problem. This is confirmed by the shape of the Pareto Front generated by the multi-objective optimization algorithms. However, there is a lack of research focused on choosing one of the solutions in the generated Pareto Front. In ref. [11], different Multi-Criteria Decision-Making (MCDM) algorithms have been implemented and compared in order to find the best one for choosing only one of the solutions after the optimization process. However, ref. [11] highlighted an issue with all of the MCDM algorithms, which was that when the weight of the spectral efficiency was even slightly higher than that of the energy efficiency, the algorithms almost always chose the solution with the highest value of SE possible.

The objective of this paper is to propose a data transformation technique that transforms the shape of the Pareto Front so that the decision-making algorithms are able to explore the whole PF for different values of criteria weights. More specifically, the transformation proposed is the Box–Cox transform, and different simulations were run with different values of the parameter λ, in order to find the best value for it. Moreover, after the transformation of the Pareto Front, four different MCDM algorithms were implemented and compared, with the goal of analyzing their behavior and the differences between them after the common issue for all of them had been solved.

The rest of this paper is organized as follows: Section 2 presents the problem statement, including the mathematical definition of the problem, the introduction to the MCDM algorithms, and the issues they have previously exhibited when implemented in the spectral and energy efficiency trade-off of Massive MIMO. Section 3 describes the proposed decision-making method, which includes the Box–Cox transformation as its first step. Section 4 defines the simulations run for this paper and summarizes the results of these simulations.

## 2. Problem Statement

### 2.1. Problem Definition

In the introduction of this paper, it is explained that Massive MIMO systems should be able to achieve high values of spectral and energy efficiency. However, these parameters contradict each other; therefore, maximizing them at the same time creates a multi-objective optimization problem, which is expressed below [8]:

(1) $$P:max\left\{\begin{array}{l} {µ}_{SE}=K{log}_{2}\;\left\{1+\left(\frac{\rho L}{K}\right)\left[1+\ln\left(\frac{N_{t}}{L}\right)\right]\right\}\\ {µ}_{EE}=\frac{K{log}_{2}\;\left\{1+\left(\frac{\rho L}{K}\right)\left[1+\ln\left(\frac{N_{t}}{L}\right)\right]\right\}}{\rho +P_{1}+LP_{2}} \end{array}\right.$$

The efficiencies calculated using the above formulas are dependent on a number of different parameters. The maximum number of antennas available in a Massive MIMO system ($N_{t}$) and the number of users that can be served by a single antenna (K) are values defined by the specific MIMO system that is being used, so these values cannot be changed by the operators according to their use case. In Formula (1), the total power consumption is calculated as the sum of the transmit power and the circuit power consumption. The circuit power consumption is also dependent on the specific MIMO system and includes the power needed for the operation of all the different equipment included in a transmission in this system, as expressed in Formula (2):

(2) $$P_{1}+LP_{2}$$

where $P_{1}=2P_{syn}+P_{LNA}+P_{mix}+P_{IFA}+{P_{filr}+P}_{ADC}$ and $P_{2}=P_{DAC}+P_{mix}+P_{filt}$. The powers mentioned in these formulas are the power consumption for the frequency synthesizer ($P_{syn})$, the low-noise amplifier ($P_{LNA})$, the mixer ($P_{mix})$, the intermediate-frequency amplifier ($P_{IFA}$), the active filters at the receiver side ($P_{filr}$), the analog-to-digital converter at the BS ($P_{ADC})$, the digital-to-analog converter ($P_{DAC}$), and the active filters at the transmitter side ($P_{filt}$) [8,12,13].

Two parameters that can be changed by the operator and that serve as the variables in this simulation model are the transmit power (ρ) and the number of active antennas (L), whose constraints are as follows:

(3) $$\rho_{min}<\rho <\rho_{max}$$

(4) $$1<L<N_{t}$$

$\rho_{min}$ and $\rho_{max}$ are the minimum and the maximum values that the transmit power can have, while the number of selected antennas can have a value between 1 and the number of available antennas, which is $N_{t}$. The maximum values of both of these variables achieve the highest spectral efficiency possible, but at the same time they increase the energy consumption significantly. Moreover, if transmitting devices operate constantly with high values of power, their lifespan decreases, and this is directly linked to higher financial costs for the mobile operator. Also, high energy consumption contradicts the global initiatives on green networking, which aim to reduce the environmental impact of mobile communication networks. Therefore, mobile operators cannot use the maximum values for the transmit power and the number of active antennas, but they need to choose these values carefully to achieve a trade-off between spectral and energy efficiency, according to their use cases.

### 2.2. Multi-Criteria Decision-Making Algorithms

Decision-making algorithms were first introduced in the 1950s and have been useful across a wide variety of fields of study, including multi-objective optimization [14]. Often researchers and engineers are faced with the requirement to maximize or minimize two or more parameters that contradict each other, and they use different multi-objective optimization techniques to address this issue. As mentioned earlier, the optimization algorithms generate a Pareto Front with the non-dominated solutions, but it is still the responsibility of the decision-maker to choose a specific solution in this PF. MCDM algorithms are designed to help decision-makers with their choice. They are simple and do not consume a lot of resources, but they require a defined set of weights for each objective, which depends on the use case. This means that the algorithms contain uncertainties and are affected by the subjectivity of the user [15].

In ref. [11] and in this paper, four of the most popular decision-making algorithms have been implemented for the spectral and energy efficiency trade-off in Massive MIMO systems: Simple Additive Weighting (SAW), Technique for Order of Preference by Similarity to Ideal Solution (TOPSIS), Preference Ranking Organization Method for Enrichment Evaluations (PROMITHEE), and the Vlse Kriterijumska Optimizacija Kompromisno Resenje (VIKOR) algorithm. The input for all algorithms is the decision matrix comprised of the values of the objectives for each solution in the Pareto Front and the criteria weights. The algorithms rank these solutions according to different requirements. SAW and TOPSIS normalize the decision matrix and then perform the ranking based on the weighted sum of the objectives and the distances from the ideal positive and negative points, respectively [16,17]. PROMITHEE ranks the solutions by calculating the differences between each pair of them regarding each of the objectives and using preference functions to transform them into a preference degree, a number between 0 and 1 [18]. The VIKOR algorithm focuses on the ranking, by proposing a compromise solution based on the estimated ideal solution and the regret (R) value of each alternative [19].

In ref. [11], it was observed that all four of the MCDM algorithms had an issue when they were implemented in the Pareto Front generated by the optimization algorithms. The simulation results, summarized in Table 1, showed that when the spectral efficiency had a higher weight than the energy efficiency, the algorithms almost always chose the solution with the highest value of the spectral efficiency objective, without considering the value of the weights. Despite different algorithms performing differently, they all left a major part of the Pareto Front unexplored.

After examining the part of the graph where there were no solutions chosen, it became evident that this region corresponded to the convex portion of the graph. On the other hand, all the solutions were focused on the concave part of the Pareto Front. In ref. [20], the authors study the multi-objective optimization based on weights and they explain the reason why it does not perform well for both possible shapes of the Pareto Front graph at the same time. They rotate the coordinate system of the graph according to the set of weights for the objectives and try to find the minimum or maximum point in the resulting graph for minimization or maximization issues, respectively.

**Figure 1:**
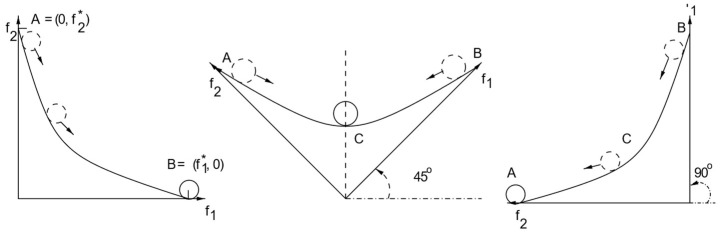
Convex Pareto Front. All Pareto solutions are stable minimum when the coordinate system rotates: 0°, 45°, and 90° [19].

**Figure 2:**
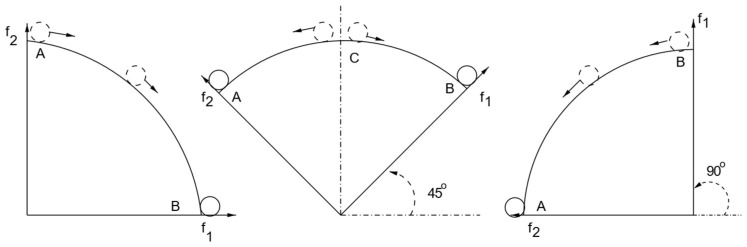
Concave Pareto Front. All Pareto solutions are unstable minimum, except the two points on both ends when the coordinate system rotates: 0°, 45°, and 90° [19].

Figure 1 shows that when rotating a convex graph, all of the Pareto Solutions are a stable minimum for a specific rotation, which means that different solutions will be chosen for different sets of weights. However, Figure 2 shows that when the shape of the graph is concave there is no stable minimum, despite the rotation, so the solution chosen is always one of the two extreme points. This proves that minimization based on weights is not suitable for concave graphs [20]. Based on a similar logic, it can be concluded that the maximization problem based on weights cannot be implemented in a convex graph. This explains the issue described earlier and in more detail in ref. [11], where the convex part of the Pareto Front is left unexplored by the decision-making algorithms. Therefore, in this paper, we propose transforming the Pareto Front with a transformation that changes its shape into a concave graph before implementing the decision-making algorithms.

## 3. Proposed Decision-Making Method

### 3.1. Power Transformation of Data

Data transformation is a technique used to simplify the structure of the data so that they are more suitable for analysis and for use in different fields of science and life. It has been used for many decades with different objectives, including stabilizing a variance, inducing a particular type of distribution, improving the normality of data, etc. [21,22]. It continues to be used widely today, as the amount of data grows rapidly. One of the most important data transformation families is the Box–Cox transformation, which is a power transformation method first introduced in 1964. The Box–Cox transformation uses a parameter λ to transform a variable y with positive values using the following formula [23]:

(5) $$y=\left\{\begin{array}{c} \frac{y^{\lambda }-1}{\lambda }\;for\;\lambda \neq 0\\ \log\left(y\right)\;for\;\lambda =0 \end{array}\right.$$

The Box–Cox transformation converges to a simple log transformation when the λ parameter is equal to 0, including the log transformation in the power transformation family. One of the main characteristics of power transformation is that the transformation is convex when λ > 1 and concave when λ < 1 [21]. Considering the fact that the maximization process based on weights can be used successfully only on concave-shaped graphs, in this paper we propose implementing the Box–Cox transformation with values of λ smaller than 1.

### 3.2. Pareto Front Transformation

**Figure 3:**
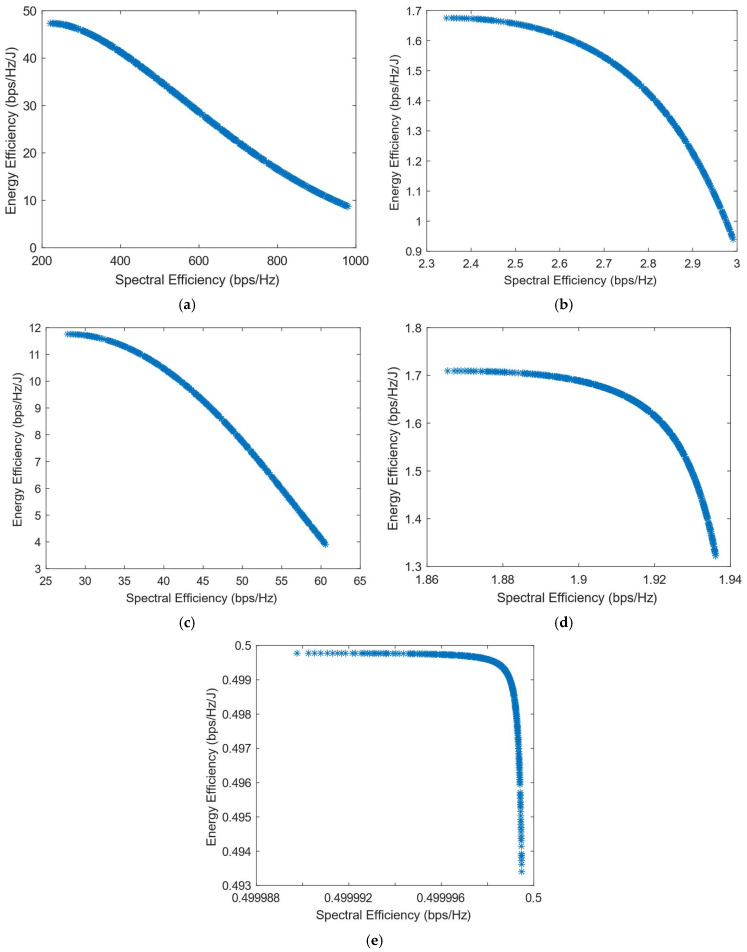
The shape of the Pareto Front after using the Box–Cox transformation. (**a**) The original Pareto Front; (**b**) the Box–Cox transform with λ = 0 (log transform); (**c**) the Box–Cox transform with λ = 0.5; (**d**) the Box–Cox transform with λ = −0.5; (**e**) the Box–Cox transform with λ = −2.

Previously, it was shown that the decision-making algorithms implemented in the generated Pareto Front had a major flaw: they did not explore the convex part of the Pareto graph. This means that none of the points in the convex part of the graph was chosen as the most suitable solution, despite the different weights used for spectral and energy efficiency. Therefore, in this paper, we propose using the Box–Cox transformation with λ < 1 to transform the Pareto Front before implementing the decision-making algorithms. The transformed Pareto Front is shown in Figure 3. It can be seen that for all the chosen values of the λ parameter which are smaller than 1, the shape of the graph is concave. Taking into consideration the new shape of the PF, we expect the decision-making algorithms to choose solutions throughout the available points for different values of weights.

## 4. Simulation Results and Discussion

### 4.1. Simulation Definition

In this paper, the simulations are run using a Pareto Front generated by the NSGA-II (Non-Dominated Sorting Genetic Algorithm-II) algorithm, according to the simulation parameters in ref. [11]. In ref. [11], it was shown that both evolutionary optimization algorithms, NSGA-II and MOPSO (Multi-Objective Particle Swarm Optimization), were successful in generating a Pareto Front close to the ideal one when they were used with the number of individuals per generation set to 400 and the maximum number of generations set to 300. Therefore, both Pareto Fronts can be used interchangeably. The next step in the simulation is the transformation of the Pareto Front in MATLAB, version R2017b, according to the Box–Cox transform described in Section 3. For this paper, the transformation is carried out using four different values for the λ parameter: 0 (the log transformation), −0.5, 0.5, and −2, so that the most suitable one for this specific problem can be chosen. After the transformation of the Pareto Front, for each transformed graph according to the value of λ, the decision-making process is run with four MCDM algorithms, as explained in Section 2: SAW, TOPSIS, PROMITHEE, and VIKOR. For the implementation of the decision-making algorithm, the code uploaded by the author in MATLAB Central File Exchange as referenced in [24] is utilized. Moreover, the simulations are run using different parameters for the algorithms, specifically four normalization methods for SAW and TOPSIS: Max, Sum, Vector, and Max–Min [25]; three preference functions for PROMITHEE: V-shape, Linear, and Gauss [26]; and three forms for the VIKOR algorithm: by consensus, by veto, and by majority rule. The goal of the simulations is to prove that the Box–Cox transformation is successful in solving the issue presented in ref. [11], and also to analyze and compare the behavior of the MCDM algorithms when they are implemented after the Pareto Front transformation.

### 4.2. Simulation Results for Log Transformation (λ = 0)

**Figure 4:**
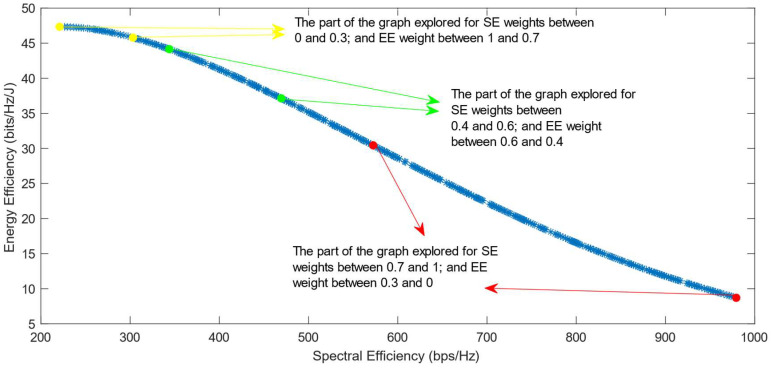
The simulation results for λ = 0: SAW algorithm implemented with Max, Sum and Vector normalization.

**Figure 5:**
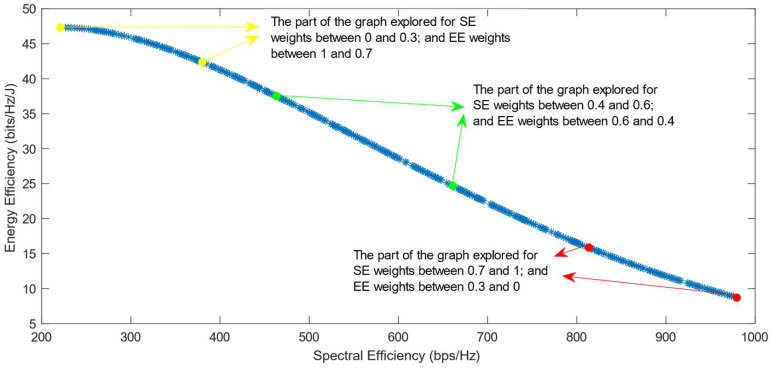
The simulation results for λ = 0: SAW algorithm implemented with Max–Min normalization.

The following figures show the solutions chosen in the Pareto Front from the decision-making process with different combinations of weights, after the PF has been transformed using the log transformation or the Box–Cox transformation when λ = 0. Figure 4 and Figure 5 show the results when the SAW decision-making algorithm is used. The results after using the normalization methods Max, Sum, and Vector are summarized in Figure 4 and the results obtained with the Max–Min normalization during the decision-making process are shown in Figure 5. It can be seen that in both cases, the whole Pareto Front has been explored, both the convex and concave parts, and there are different solutions chosen for different sets of weights. However, in the first figure, it can be observed that the convex part of the graph is explored in a small interval of weights, specifically for SE weights varying from 0.7 to 1, meaning that the solutions chosen in this part are distant from each other. Therefore, the concave part of the graph is still better explored than the convex part. This issue is minimized in the second figure, when the Max–Min normalization is used. In this case, parts of the graph with different shapes are explored almost in the same way, but there are still parts of the PF in its convex part that are not fully explored. For example, the solution chosen for the pair of weights (0.6; 0.4) is still far from the solution chosen for weights (0.7; 0.3), which is also far from the solution chosen for weights (0.8; 0.2); thus, even though this normalization method achieves better results, it still does not guarantee an optimized distribution of the solutions.

**Figure 6:**
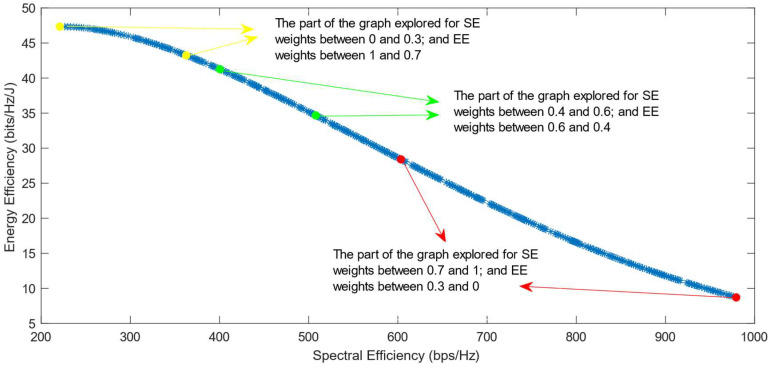
The simulation results for λ = 0: TOPSIS algorithm implemented with Max, Sum, and Vector normalization.

**Figure 7:**
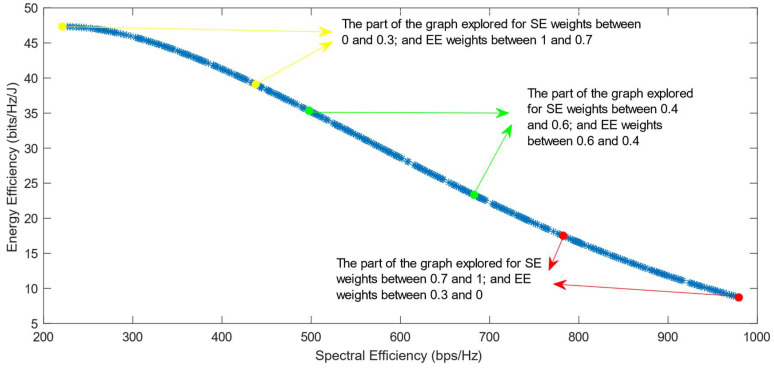
The simulation results for λ = 0: TOPSIS algorithm implemented with Max–Min normalization.

**Figure 8:**
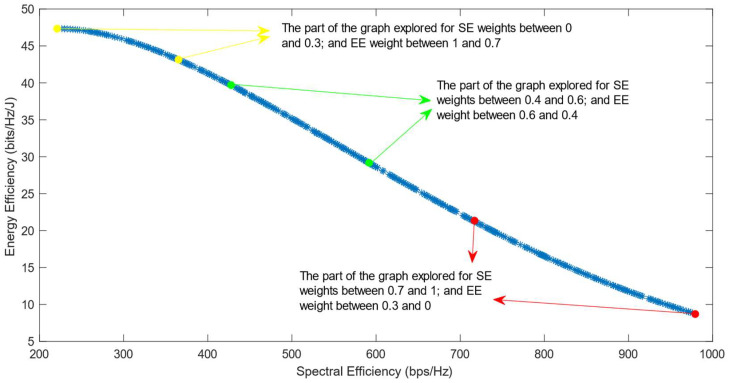
The simulation results for λ = 0: PROMITHEE algorithm implemented with V-Shape and Gauss preference function.

Figure 6, Figure 7 and Figure 8 show that similar results are generated when the TOPSIS and PROMITHEE decision-making algorithms are used. However, when implementing the TOPSIS algorithm with the Max–Min normalization method, it ensures a better distribution of the solutions chosen for different weights. The PROMITHEE algorithm performs well when used with the V-shape or Gauss preference function, which means it chooses solutions throughout the whole graph, although small parts of the graph remain unexplored. However, this algorithm cannot be used with a linear preference function in this case. As the simulations demonstrate, it chooses the point with the highest value of SE and the lowest value of EE, despite changing the weight of each of these objectives.

**Figure 9:**
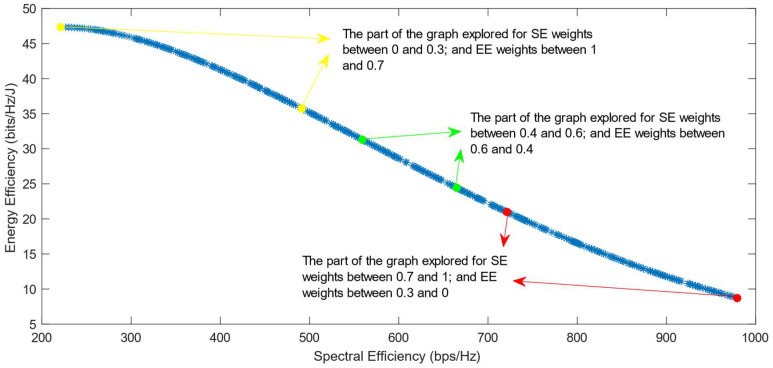
The simulation results for λ = 0: VIKOR algorithm.

Out of all the simulations run with different DM algorithms and different specific parameters for each of them, the VIKOR algorithm is less dependent on the parameters of the algorithm itself. It performs well in all three of its forms: by consensus, by veto, or by majority rule. As can be seen in Figure 9, all parts of the graph are explored in the same way and the solutions chosen for different sets of weights are almost equally distant from each other, achieving an optimized distribution.

### 4.3. Simulation Results for Box–Cox Transformation with λ = −0.5

**Figure 10:**
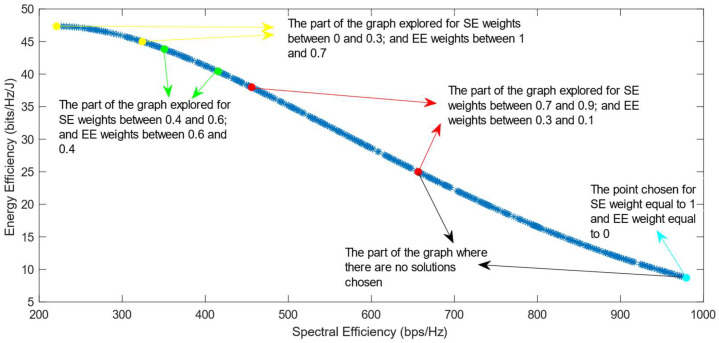
The simulation results for λ = −0.5: SAW and TOPSIS algorithms implemented with Max, Sum, and Vector normalization.

**Figure 11:**
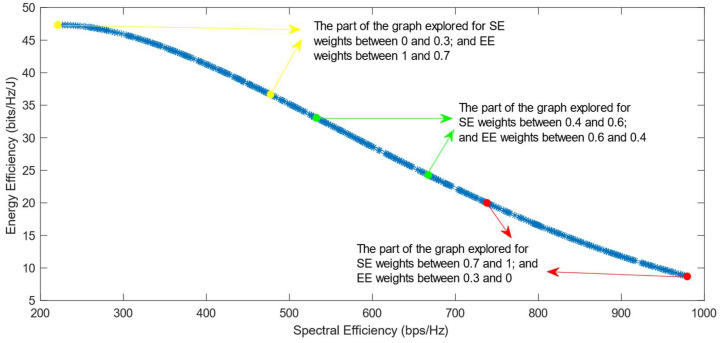
The simulation results for λ = −0.5: SAW and TOPSIS algorithms implemented with Max–Min normalization.

**Figure 12:**
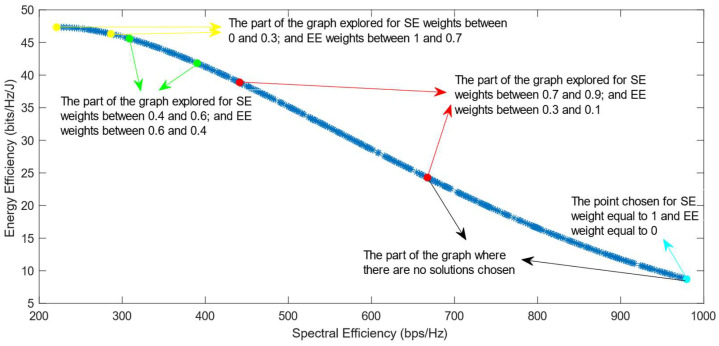
The simulation results for λ = −0.5: PROMITHEE algorithm implemented with V-Shape preference function.

**Figure 13:**
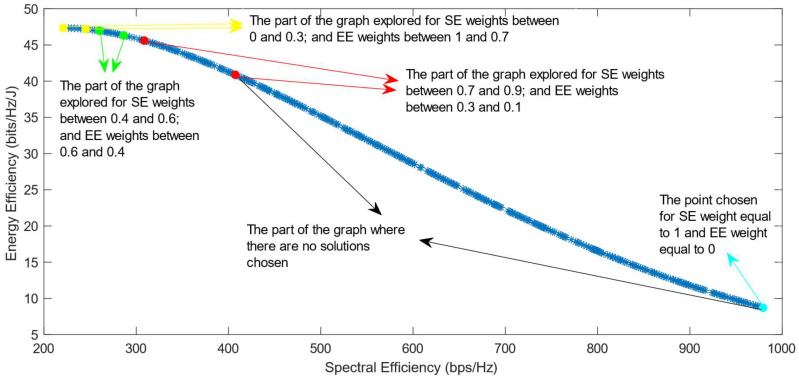
The simulation results for λ = −0.5: PROMITHEE algorithm implemented with Gauss preference function.

**Figure 14:**
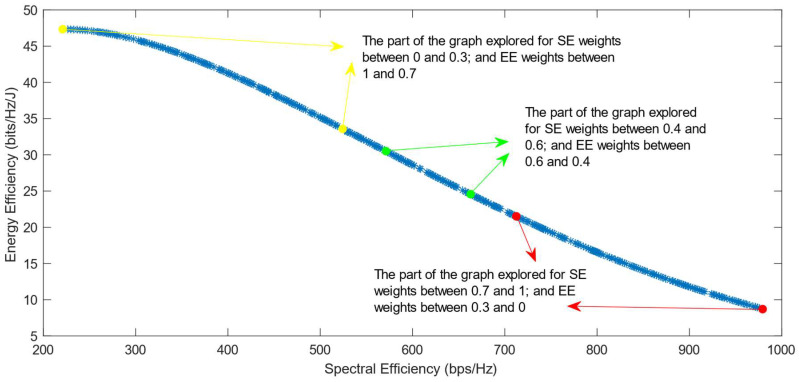
The simulation results for λ = −0.5: VIKOR algorithm.

Figure 10, Figure 11, Figure 12, Figure 13 and Figure 14 show the simulation results when using the different DM algorithms after the transformation of the Pareto Front with the Box–Cox transformation for λ = −0.5. In Figure 3d, it can be observed that the Pareto Front shape after this transformation is concave, but there are parts of it that almost exhibit a linear shape. This characteristic of the transformed PF explains the reason why there are major parts of the graph in its convex part where there are no solutions chosen, as can be seen in Figure 10, Figure 12 and Figure 13. This proves that the Box–Cox transformation with λ = −0.5 does not fully solve the original issue highlighted in ref. [11], where there is no transformation used in the Pareto Front. The simulations show that the issue is solved when, after this transformation, the DM algorithms used are VIKOR or SAW and TOPSIS with Max–Min normalization.

### 4.4. Simulation Results for Box–Cox Transformation with λ = 0.5

**Figure 15:**
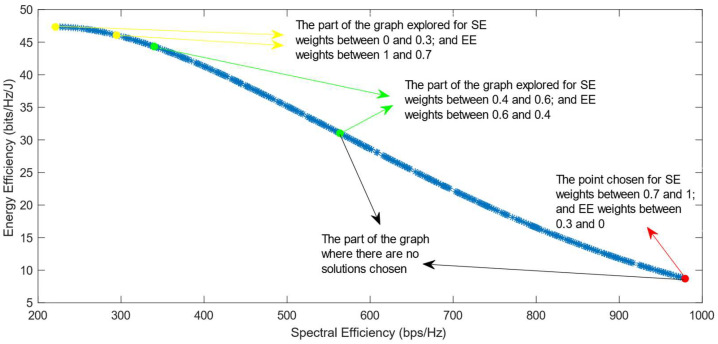
The simulation results for λ = 0.5: SAW algorithm implemented with Max, Sum, and Vector normalization.

**Figure 16:**
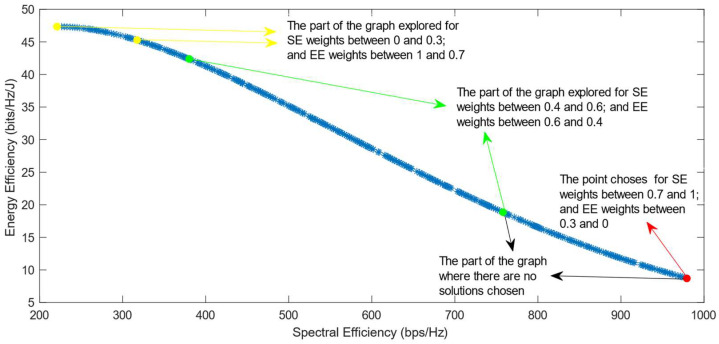
The simulation results for λ = 0.5: SAW algorithm implemented with Max–Min normalization.

**Figure 17:**
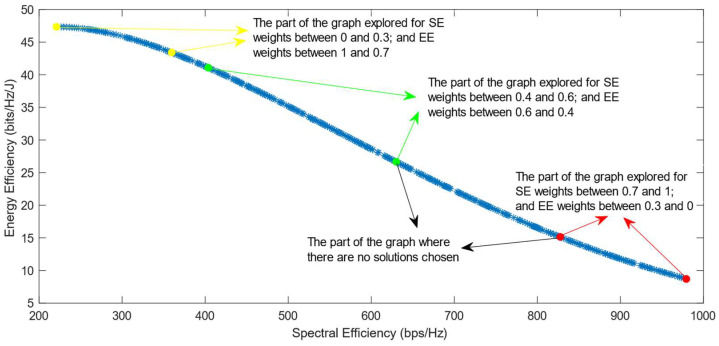
The simulation results for λ = 0.5: TOPSIS algorithm implemented with Max, Sum, and Vector normalization.

**Figure 18:**
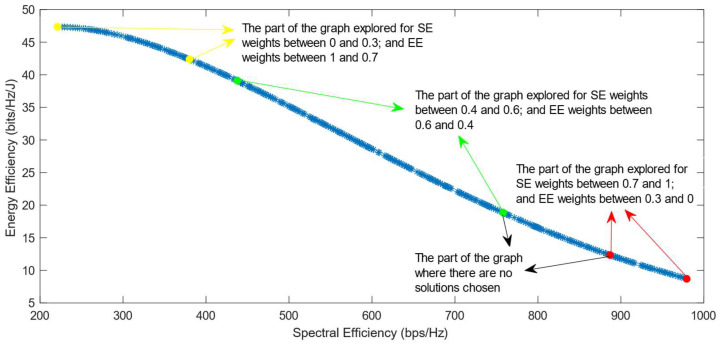
The simulation results for λ = 0.5: TOPSIS algorithm implemented with Max–Min normalization.

**Figure 19:**
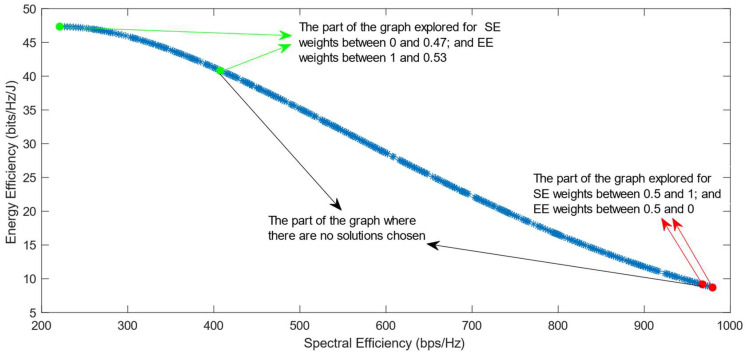
The simulation results for λ = 0.5: PROMITHEE algorithm.

**Figure 20:**
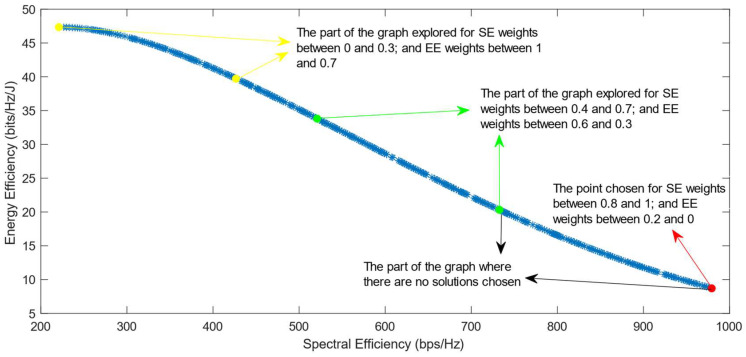
The simulation results for λ =0.5: VIKOR algorithm.

The Box–Cox transformation with λ = 0.5 further emphasizes the problem shown in the previous paragraph, where the transformation is used with λ = −0.5. As can be seen in Figure 15, Figure 16, Figure 17, Figure 18, Figure 19 and Figure 20, despite the DM algorithm, this transformation does not solve the original issue with the decision-making process, leaving a large part of the Pareto Front unexplored, meaning that there are no solutions chosen in these parts for any of the values of the SE and EE weights.

### 4.5. Simulation Results for Box–Cox Transformation with λ = −2

**Figure 21:**
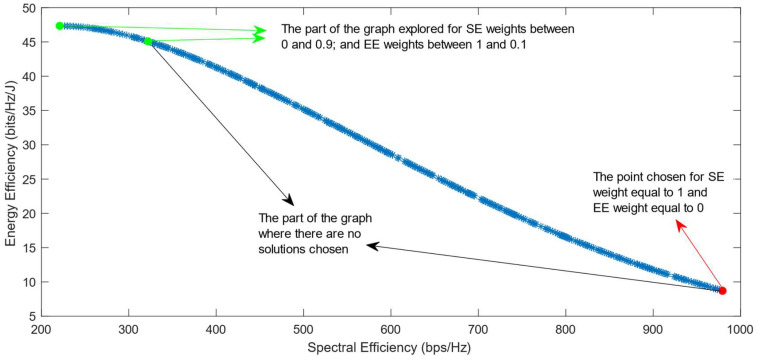
The simulation results for λ = −2: SAW and TOPSIS algorithms implemented with Max, Sum, and Vector normalization.

**Figure 22:**
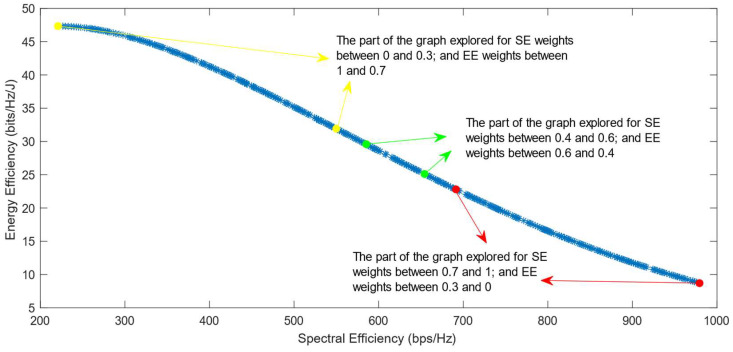
The simulation results for λ = −2: SAW and TOPSIS algorithms implemented with Max–Min normalization.

**Figure 23:**
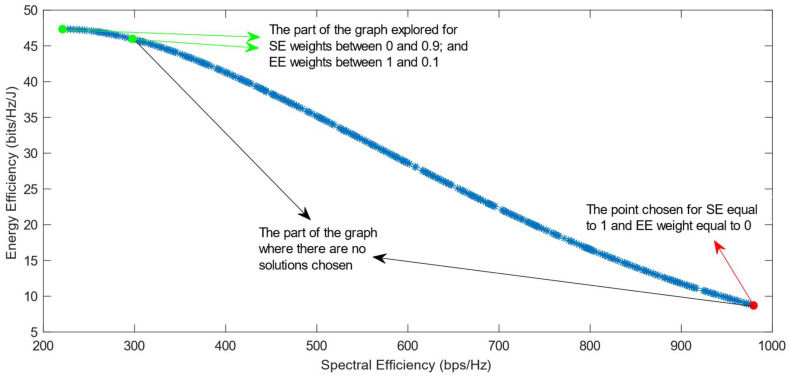
The simulation results for λ = −2: PROMITHEE algorithm implemented with V-Shape preference function.

**Figure 24:**
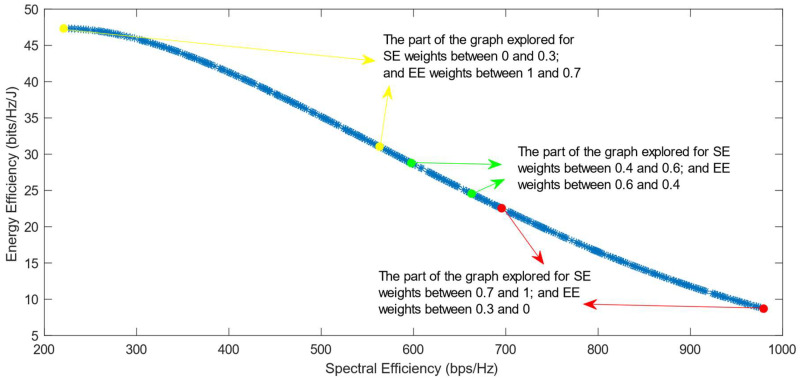
The simulation results for λ = −2: VIKOR algorithm.

Figure 21, Figure 22, Figure 23 and Figure 24 show the simulation results when using the proposed transformation method with λ = −2. In this case, the implementation of SAW and TOPSIS with Max, Sum, and Vector normalization methods leaves a large part of the Pareto Front unexplored, as shown in Figure 21. The Max–Min normalization method, as well as the VIKOR algorithm, manage to solve this issue and explore almost the whole graph, but the simulations show that the solutions chosen for the weights (0.9, 0.1) and (1,0) are distant from each other, which leaves a small part of the graph still unexplored. Regarding the PROMITHEE algorithm, simulations show that it is not suitable for use after the Box–Cox transformation with λ = −2 in this decision-making problem. Figure 23 shows the results generated by the implementation of PROMITHEE with the V-shape preference function, while the Linear and Gauss preference functions cannot be used, as they choose only the extreme points of the graph and do not explore the Pareto Front at all.

### 4.6. Summarization of the Simulation Results

All the simulation results explained in detail in the previous paragraphs are presented in Table 2.

## 5. Conclusions

The spectral and energy efficiency trade-off in Massive MIMO systems is a multi-objective optimization problem that is highly relevant to rapidly evolving mobile communication networks. There has been much research focused on using the evolutionary optimization algorithms for solving this issue, but this paper is focused in choosing a specific solution in the Pareto Front generated by these algorithms. The Box–Cox transformation is proposed as the first step before the implementation of the MCDM algorithms so that the complex shape of the Pareto Front is transformed into a concave shape. This ensures that the decision-making algorithms explore the whole Pareto Front when choosing solutions based on different values of the objectives’ weights. It is shown by the simulations that the best value for the λ parameter is 0, also known as the log transformation. Using this value for the transformation solves the issue of the unexplored convex part of the Pareto Front for all MCDM algorithms, except for the PROMITHEE algorithm used with a linear preference function. Moreover, it is demonstrated that the decision-making algorithms with the best performances are the SAW and TOPSIS when they are combined with the Max–Min normalization technique, and the VIKOR algorithm. The solutions chosen by these have the best distribution, as they have an almost equal distance from each other. It is also proven by the simulations that the performance of the VIKOR algorithm is not affected by the parameters of the algorithm itself, as its behavior did not change despite the form used: by consensus, by veto, or by majority rule. These algorithms function properly even with different values of λ, a condition which proves to be problematic for other algorithms. To sum up, in this paper it is shown that MCDM algorithms such as VIKOR, SAW, and TOPSIS are suitable for choosing a specific solution in the Pareto Front after it is transformed by the log algorithm. They can help the decision-maker choose according to his specific use case and guarantee the trade-off between the spectral and energy efficiency in Massive MIMO systems.

Further research done on the decision-making proposed in this paper can establish its applicability and the validity in real-world scenarios. Moreover, future work can focus on other transformation techniques that change the shape of the Pareto Front into a concave shape and on the comparison of these techniques with the Box–Cox transformation, so that the best one is chosen based on their disadvantages and their benefits.

## Figures and Tables

**Table 1:** Simulation results for different combinations of weights in the same Pareto Front [11].

Weights		SAW		TOPSIS		PROMITHEE		VIKOR	
*W_1_ (SE)*	*W_2_ (EE)*	*Spectral Efficiency* *(bps/Hz)*	*Energy Efficiency* *(bps/Hz/J)*	*Spectral Efficiency* *(bps/Hz)*	*Energy Efficiency* *(bps/Hz/J)*	*Spectral Efficiency* *(bps/Hz)*	*Energy Efficiency* *(bps/Hz/J)*	*Spectral Efficiency* *(bps/Hz)*	*Energy Efficiency* *(bps/Hz/J)*
***0.9***	***0.1***	979.33	8.71	979.33	8.71	979.33	8.71	979.33	8.71
***0.8***	***0.2***	979.33	8.71	979.33	8.71	979.33	8.71	979.33	8.71
***0.7***	***0.3***	979.33	8.71	979.33	8.71	979.33	8.71	979.33	8.71
***0.6***	***0.4***	979.33	8.71	979.33	8.71	979.33	8.71	672.6	23.94
***0.58***	***0.42***	979.33	8.71	979.33	8.71	979.33	8.71	659.6	24.76
***0.55***	***0.45***	979.33	8.71	979.33	8.71	979.33	8.71	638.67	26.11
***0.53***	***0.47***	393.76	41.64	437.72	39.1	979.33	8.71	624.82	26.99
***0.5***	***0.5***	359.08	43.44	402.05	41.18	286.53	46.32	466.4	37.33
***0.47***	***0.53***	329.76	44.78	376.76	42.55	282.1	46.44	441.36	38.89
***0.45***	***0.55***	317.4	45.28	369.5	42.93	282.1	46.44	427.88	39.7
***0.42***	***0.58***	303.28	45.79	352.45	43.76	279.34	46.52	407.37	40.89
***0.4***	***0.6***	297.7	45.98	344.49	44.13	279.34	46.52	393.76	41.64
***0.3***	***0.7***	263.81	46.89	317.4	45.28	277.59	46.57	317.4	45.28
***0.2***	***0.8***	245.17	47.17	293.01	46.13	245.18	47.19	277.6	46.57
***0.1***	***0.9***	232.94	47.3	265.3	46.86	226.53	47.33	245.18	47.19
***0.05***	***0.95***	226.53	47.33	252.47	47.09	226.53	47.33	232.94	47.3
***0.02***	***0.98***	220.96	47.34	236.67	47.28	220.96	47.34	226.53	47.33

**Table 2:** Outline of all the simulation results.

	λ = 0	λ = −0.5	λ = 0.5	λ = −2
**SAW**	- For Max, Sum, and Vector normalization, the solutions chosen in the convex part are far from each other. - For Max–Min normalization, small parts of the PF remain in its convex part that are not fully explored.	- For Max, Sum, and Vector normalization, the convex part of the PF is mostly unexplored. - For Max–Min normalization, there are solutions chosen in all parts of the PF with a good distribution.	- For all normalization methods, the convex part of the graph is not fully explored.	- For Max, Sum, and Vector normalization, most of the PF is left unexplored. - For Max–Min normalization, there are small parts with convex shape in the PF where there are no solutions chosen.
**TOPSIS**	- For Max, Sum, and Vector normalization, the solutions chosen in the convex part are far from each other. - For Max–Min normalization, the solutions chosen are well-distributed throughout the whole PF.	- For Max, Sum, and Vector normalization, the convex part of the PF is mostly unexplored. - For Max–Min normalization, the whole PF is explored with a good distribution of solutions.	- For all normalization methods, the convex part of the graph is not fully explored.	- For Max, Sum, and Vector normalization, most of the PF is left unexplored. - For Max–Min normalization, there are small parts with convex shape in the PF where there are no solutions chosen.
**PROMITHEE**	- For V-shape and Gauss, there are solutions chosen in all parts of the PF with small parts of it left unexplored. - For the Linear preference function, it chooses only the extreme points in the PF.	- For V-shape and Gauss preference function, there are major parts of the PF where no solution is chosen. - For the Linear preference function, it chooses only the solution with the highest SE possible.	- For all preference functions, there are no solutions chosen in the convex part of the PF.	- For V-shape preference function, most of the PF is left unexplored. - For the Linear and Gauss preference function, only the extreme points of the graph are chosen as solutions.
**VIKOR**	- It explores the whole PF with a good distribution of solutions in all three of its forms.	- It explores all parts of the PF with a good distribution of solutions in all three of its forms.	- There are parts of the PF with the convex shape that are left unexplored.	- It explores the whole PF, although there are small parts with convex shape where there are no solutions chosen.

## Data Availability

All of the original data presented in the study are included in the article; further inquiries can be directed to the corresponding author.

## Abbreviations

The following abbreviations are used in this manuscript:

MIMO	Multiple Input, Multiple Output
MCDM	Multi-Criteria Decision-Making
SAW	Simple Additive Weighting
TOPSIS	Technique for Order of Preference by Similarity to Ideal Solution
PROMITHEE	Preference Ranking Organization Method for Enrichment Evaluations
VIKOR	Vlse Kriterijumska Optimizacija Kompromisno Resenje
SE	Spectral Efficiency
EE	Energy Efficiency
PF	Pareto Front
NSGA-II	Non-Dominated Sorting Genetic Algorithm-II
MOPSO	Multi-Objective Particle Swarm Optimization
